# Debulking of a large right heart mass in a cancer patient using the Angiovac system

**DOI:** 10.1093/ehjcr/ytaa432

**Published:** 2020-12-01

**Authors:** Mark Lebehn, Polydoros N Kampaktsis, Sanjum Sethi, Nadira Hamid

**Affiliations:** Columbia University Medical Center, New York-Presbyterian Hospital, 177 Fort Washington Avenue, New York, NY 10032, USA

A 55-year-old woman with metastatic pancreatic adenocarcinoma on chemotherapy presented with one week of fatigue, dyspnoea on exertion, and lower extremity oedema. Computed tomographic angiography of chest revealed right lower lobe pulmonary embolus and filling defects in the right atrium (*Panel A*). Blood cultures were negative and inflammatory markers were normal. Transthoracic echocardiogram revealed a large, irregular, mobile mass in the right atrium attached to and abutting the tricuspid valve with preserved right ventricular function (*Panel B*). Lower extremity duplex ultrasound was negative for deep vein thrombosis. Differential diagnosis included thrombus, non-bacterial thrombotic endocarditis, and cardiac metastasis. A multi-disciplinary discussion was held. Palliative mechanical thrombectomy was performed due to significant obstruction of flow from the right atrium to the right ventricle. Intra-procedural transoesophageal echocardiography (TEE) showed an extremely large irregular mass engulfing right atrium and extending from the tricuspid valve to superior vena cava junction around the patient’s chemotherapy catheter (*Panels C* and *D* and *Videos 1* and 2) Smaller masses were also seen on the mitral valve (*Panels E* and *F* and *Video 3*). Under echocardiographic guidance, the Angiovac cannula (*Panel G*) was used to perform thrombectomy and debulk significant portion of the right-sided mass (*Panel H*). Histopathology confirmed thrombus with no evidence of malignant cells. She was continued on intravenous therapeutic heparin and discharged on subcutaneous heparin. Intra-procedural TEE was critical to visualize placement of the cannula to avoid thrombus embolization. This case illustrates a massive thrombus successfully debulked using a catheter-based approach.

**Figure:**
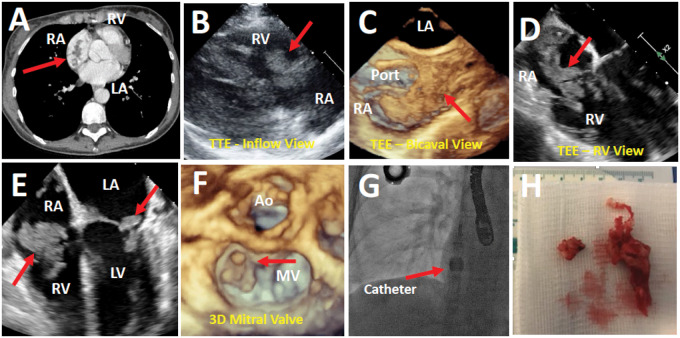


**Consent:** The authors confirm that written and verbal consent for submission and publication of this case report, including image(s) and associated text has been obtained from patient in line with COPE guidance.

